# Host Factor Nucleoporin 93 Is Involved in the Nuclear Export of Influenza Virus RNA

**DOI:** 10.3389/fmicb.2018.01675

**Published:** 2018-07-24

**Authors:** Yuri Furusawa, Shinya Yamada, Yoshihiro Kawaoka

**Affiliations:** ^1^Division of Virology, Department of Microbiology and Immunology, The Institute of Medical Science, The University of Tokyo, Tokyo, Japan; ^2^Department of Pathobiological Sciences, School of Veterinary Medicine, University of Wisconsin-Madison, Madison, WI, United States; ^3^Department of Special Pathogens, International Research Center for Infectious Diseases, The Institute of Medical Science, The University of Tokyo, Tokyo, Japan

**Keywords:** influenza A virus, host factor, Nucleoporin 93, nuclear export, A549

## Abstract

Influenza virus replication relies on the functions of host factors. In our previous study, we identified host factors involved in virus replication and began analyses of their roles in this process. In this study, we focused on Nucleoporin 93 (NUP93) and revealed its importance in influenza virus replication. NUP93 knockdown mediated by siRNAs reduced viral replication and decreased the efficiency of the early step of the viral life cycle. NUP93 did not appear to be important for virus binding, internalization, or the nuclear import of viral ribonucleoprotein (vRNP); however, in NUP93-depleted cells, viral RNA accumulated in the nucleus. These results suggest that NUP93 is involved in the nuclear export of viral RNA.

## Introduction

Influenza viruses cause acute respiratory infection, and the annual epidemics of influenza virus have an enormous impact on the global economy and public health. Once viruses infect host cells, they replicate by relying on the functions of numerous host factors. These host factors interact with the viral components and play important roles in the virus life cycle. Identifying host factors involved in virus replication and determining their roles in this process will help us to better understanding the virus life cycle. Furthermore, host factors required for virus replication can be targets for the development of new antiviral drugs. Because current antiviral drugs target viral proteins that mutate rapidly, the emergence of drug-resistant viruses has become a problem. Host factor-targeting antiviral drugs are expected to be less prone to lead to the generation of drug-resistant viruses.

We previously performed a virus-host interactome screen using human embryonic kidney (HEK) 293 cells in an attempt to find targetable host factors (Watanabe et al., 2014). We identified 1,292 host proteins that interact with influenza viral proteins. Moreover, we found that 323 of these host proteins were involved in virus replication, since virus titers were modified by their siRNA-mediated downregulation. Further analyses of the precise roles of these proteins have been ongoing.

In this study, we focused on one of these 323 proteins, namely, Nucleoporin 93 (NUP93) (Grandi et al., 1997), which is a main component of the nuclear pore complex. siRNA-mediated knockdown of NUP93 reduced influenza virus replication. Although it seems likely that NUP93 would play an important role in virus replication given that influenza virus genomes are replicated in the nucleus, its precise role in the viral life cycle has remained unclear. Here, we used human lung epithelial cells (i.e., A549 cells) as a model of respiratory epithelial cells, which are target cells of influenza viruses, to explore how NUP93 takes part in the influenza viral life cycle. We show that NUP93 is important for virus replication and nuclear export of viral RNA in A549 cells. Our data thus suggested that NUP93 was involved in the early step of the viral life cycle.

## Materials and Methods

### Cells and Viruses

A549 (human lung epithelial) cells were cultured in Ham’s F-12K (Wako) supplemented with 10% FCS at 37°C in 5% CO_2_. Madin-Darby Canine Kidney (MDCK) cells were cultured in Eagle’s MEM (GIBCO) with 5% NCS at 37°C in 5% CO_2_. A/WSN/1933 (H1N1) was propagated in MDCK cells and titrated by plaque assay in MDCK cells (Tobita et al., 1975). The PB2-knockout virus encoding *Renilla* luciferase (PB2-KO/Rluc virus) was propagated and titrated in MDCK cells stably expressing the PB2 protein as described previously (Ozawa et al., 2011). All experiments were performed under biosafety level 2 (BSL2) conditions.

### siRNA Treatment

Four siRNAs targeting different regions of the human NUP93 gene were purchased from QIAGEN (Flexi Tube siRNA). Hs_NUP93_6, Hs_NUP93_7, Hs_NUP93_8, and Hs_NUP93_9 were used as siNUP93 #1, siNUP93 #2, siNUP93 #3, and siNUP93 #4, respectively. Allstars Negative Control siRNA (QIAGEN) served as a negative control siRNA (siNC). The siRNA targeting the influenza virus nucleoprotein (NP) gene (GGA UCU UAU UUC UUC GGA GUU) (Ge et al., 2003) was purchased from Sigma–Aldrich. A549 cells were transfected twice with 25 nM (final concentration, 50 nM) of siRNA duplexes using RNAiMAX Reagent (Invitrogen) with a 24 h interval between transfections. At 24 h after the second transfection, cell viability was measured using the CellTiter-Glo assay system (Promega) according to the manufacturer’s instructions. To assess influenza viral replication, three parallel sets of siRNA-transfected cells were infected with WSN virus at a multiplicity of infection (MOI) of 0.001 at 24 h after the second siRNA transfection. The supernatants were collected at the indicated times post-infection and virus titers were determined by means of plaque assays in MDCK cells.

### Western Blotting

Cells were lysed with Tris-Glycine SDS sample buffer (Invitrogen), heated for 10 min at 95°C, and then subjected to SDS–polyacrylamide gel electrophoresis (PAGE). SDS–PAGE was performed on any kD Mini-PROTEAN TGX Precast Protein Gels (Bio-Rad). Proteins on SDS–PAGE gels were transferred to a polyvinylidene fluoride (PVDF) membrane for 30 min at 15 V, and the membrane was then incubated with Blocking One (Nacalai Tesque) for 30 min at room temperature. Then, the membrane was incubated with the indicated primary antibodies [rabbit anti-NUP93(Proteintech), mouse anti-β-actin(Sigma–Aldrich), or rabbit anti-WSN (R309)] for 1 h at room temperature or overnight at 4°C, followed by three washes with PBS plus Tween 20 (PBST). Finally, the membrane was incubated with secondary antibodies [sheep horseradish peroxidase (HRP)-conjugated anti-mouse IgG (GE Healthcare) or donkey HRP-conjugated anti-rabbit IgG (GE Healthcare)], for 1 h at room temperature and washed three times with PBST. Specific proteins were detected by using Chemi-Lumi One Super (Nacalai Tesque). Images were captured with the ChemiDoc Touch Imaging System (Bio-Rad).

### PB2-KO/Rluc Virus Assay

A549 cells were transfected twice with siRNA as described above. At 24 h after the second siRNA transfection, the cells were infected with the PB2-knockout WSN virus (PB2-KO/Rluc virus) at an MOI of 1; 100 μg/ml amantadine (Sigma–Aldrich) was used as a positive control. PB2-KO/Rluc virus is a replication-incompetent virus that carries a reporter gene encoding *Renilla* luciferase in place of the coding region of the viral PB2 protein. At 8 h post-infection, *Renilla* luciferase activity was measured with the *Renilla* Luciferase Assay System (Promega). This virus is not able to synthesize PB2, therefore its replication is limited and dependent on the PB2 protein supplied with the infecting vRNP complexes.

### Immunofluorescence Microscopy

A549 cells were transfected twice with siRNA as described above. At 24 h after the second siRNA transfection, the cells were infected with WSN virus at an MOI of 200 in the presence of 100 μg/ml cycloheximide (CHX; Sigma–Aldrich) to detect incoming vRNP. We used 100 nM Baffilomycin-A1 (Baf-A1; LC Laboratories) as a positive control. At 2 h post-infection, the cells were fixed with 4% paraformaldehyde (PFA), permeabilized with 0.2% Triton X-100/PBS, and then incubated with mouse anti-NP monoclonal antibody (2S-347/3) for 1 h at room temperature. After three washes with PBS, the cells were incubated with Alexa Fluor 488 goat anti-mouse IgG for 1 h at room temperature. Nuclei were stained with Hoechst 33342 (Invitrogen). Images were obtained using a Zeiss LSM780 (Carl Zeiss).

### Virus Binding and Internalization

Virus binding and internalization analyses were performed as described previously (Liu et al., 2015). As a control, cells were pretreated with 5 units/ml of sialidase from *Arthrobacter ureafaciens* (Sigma–Aldrich) at 37°C for 8 h. The control cells and siRNA-transfected cells were then incubated with WSN virus at an MOI of 10 at 4°C for 1 h and then washed with ice-cold PBS. Cell lysates were prepared with Tris-Glycine SDS sample buffer (Invitrogen). For virus internalization, cells were warmed to 37°C for 30 min after being incubated with virus at 4°C for 1 h. They were then washed with cold PBS–HCl (pH 1.3) to remove attached virus and detect only internalized virus particles. Cell lysates were also prepared to determine influenza virus matrix protein 1 (M1) levels by Western blotting with anti-WSN (R309) antibody.

### Fluorescence *in Situ* Hybridization (FISH)

FISH for influenza virus mRNA and vRNA was performed as described previously (Amorim et al., 2007; Morita et al., 2013). Digoxigenin-labeled probes against NP or M mRNA/vRNA were generated by using the DIG-RNA labeling kit (Roche). Plasmids pSPT18-NP and pSPT18-M contain cDNA copies of WSN NP and M, respectively, flanked by the T7 and SP6 promoter. To generate FISH probes specific to mRNA, plasmids pSPT18-NP and pSPT18-M, linearized by digestion with EcoRI, were used as templates for *in vitro* transcription with SP6 RNA polymerase. To generate FISH probes specific to vRNA, pSPT18-NP and pSPT18-M, linearized by digestion with SalI, were used for *in vitro* transcription with T7 RNA polymerase. A549 cells were transfected with siRNA as described above. At 24 h after the second siRNA transfection, the cells were infected with WSN virus at an MOI of 10 (250 μg/ml 5,6-dichloro-1-β-D-ribofuranosyl-benzimidazole (DRB; Sigma–Aldrich) or 5 nM Leptomycin B (LMB; Sigma–Aldrich) served as controls). At 8 h post-infection, the cells were fixed and permeabilized. The cells were then pre-hybridized for 1 h and then hybridized with the RNA probe for 16 h at 37°C. Bound probe was detected by indirect immunofluorescence with anti-digoxigenin-fluorescein, Fab fragments (Roche). Nuclei were stained with Hoechst 33342 (Invitrogen). For polyA host RNA detection, a Cy3-labeled oligo dT probe (Sigma–Aldrich) was used. siRNA-transfected A549 cells were fixed and hybridized as described above. Images were obtained using a Zeiss LSM780 (Carl Zeiss). Quantification was performed using CellProfiler software (Carpenter et al., 2006). The nuclear region was recognized based on the fluorescence intensity of the Hoechst stain. The ring region encompassing 10 pixels from the nucleus border was defined as the cytoplasmic region. The mean intensity of RNA fluorescence was detected in each nuclear and cytoplasmic region. The RNA C/N ratio, defined as the mean intensity in the Cytoplasm divided by the mean intensity in the Nucleus, was determined in 200 cells per condition.

### Statistical Analyses

Statistical analyses [one-way or two-way analysis of variance (ANOVA) followed by Dunnett’s test] were performed using GraphPad Prism software (v6.05). Statistical significance was calculated against the values in siNC-transfected cells in each experiment.

## Results

### NUP93 Is Involved in Influenza Virus Replication in A549 Cells

**FIGURE 1:**
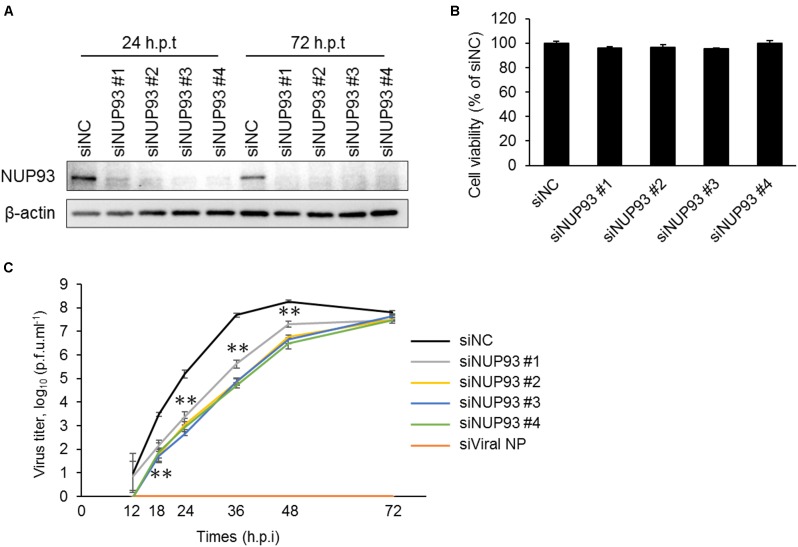
Knockdown of NUP93 leads to a reduction in influenza virus replication. A549 cells were transfected twice with negative control siRNA (siNC) or siRNAs targeting NUP93 (siNUP93). **(A)** Western blotting with NUP93 or β-actin antibody to confirm the knockdown effect. (h.p.t: hours post the second siRNA transfection) **(B)** Cell viability of siRNA-transfected cells as determined by using CellTiter-Glo. **(C)** Virus growth in siRNA-transfected cells infected with WSN virus at an MOI of 0.001. (h.p.i: hours post-infection). Means ± SD of triplicate experiments are shown in **(B)** and **(C)**. ^∗∗^*p* < 0.01 (two-way ANOVA followed by Dunnett’s test). All results are representative of at least three independent experiments.

Our previous work suggested that NUP93, one of the components of the nuclear pore complex, was important for influenza virus replication, because NUP93 knockdown led to a reduction in influenza virus replication in HEK293 cells (Watanabe et al., 2014). However, it was not known whether NUP93 was also involved in influenza virus replication in other cell types. Therefore, we examined the importance of NUP93 in virus replication using A549 (human lung epithelial) cells, because influenza viruses normally infect and propagate in respiratory epithelial cells. We downregulated NUP93 expression using four different siRNAs against NUP93 (siNUP93) that targeted different regions of the human NUP93 gene. All of these siRNAs reduced the expression of NUP93 significantly compared to the negative control siRNA (siNC), which does not target any human gene sequence (**Figure 1A** and Supplementary Figure 1). In addition, we monitored the viability of siNUP93-transfected cells and found that more than 90% of the cells remained viable at 24 h after the second siRNA transfection, the timepoint when the cells were infected with virus for subsequent experiments (**Figure 1B**). Then, to confirm the growth of virus in the NUP93-depleted cells, A549 cells were transfected with siNUP93 and infected with A/WSN/1933 (H1N1). The supernatants were collected at the indicated timepoints, and virus titers were determined by use of plaque assays. siRNA targeting viral NP (siViral NP), used as control, inhibited virus replication completely, and downregulation of NUP93 caused a significant reduction in virus growth (**Figure 1C**). Virus titers were reduced by up to two log_10_ units. In contrast, there was no significant difference at 72 h.p.i. The viral titer in siNC-treated cells reached a plateau at around 48 h.p.i and began to fall at 72 h.p.i. Although siNUP93 decreases the efficiency of viral replication, it does not inhibit viral replication completely. Moreover, the knockdown effect of siRNA might be reduced as time passed such that the viral titers in siNUP93-treated cells increased slowly but steadily, and reached the same level as that in siNC-treated cells at 72 h.p.i. These data indicate that NUP93 plays an important role in influenza virus replication not only in HEK293 cells but also in A549 cells.

### NUP93 Is Involved in the Early Steps of Influenza Virus Replication

**FIGURE 2:**
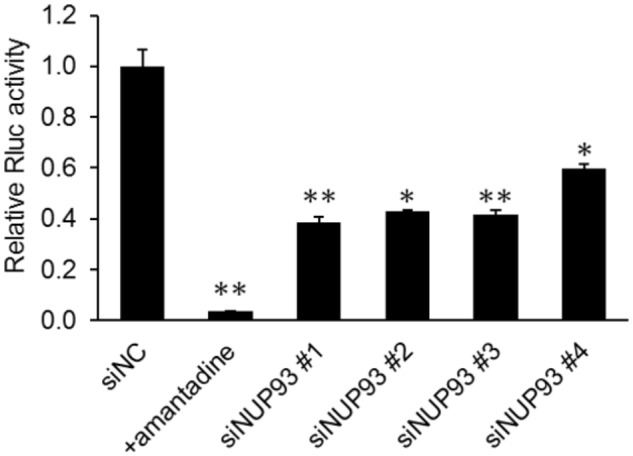
Role of NUP93 in the early steps of the influenza virus life cycle. siRNA-transfected A549 cells were infected with PB2-KO/Rluc virus at an MOI of 1. Amantadine was used as a positive control. *Renilla* luciferase activity was measured at 8 h post-infection. Values represent the means of triplicate experiments ± SD. ^∗^*p* < 0.05, ^∗∗^*p* < 0.01 (one-way ANOVA followed by Dunnett’s test).

We next examined whether NUP93 was involved in the early steps of the influenza virus life cycle. We used the PB2-knockout/*Renilla* luciferase virus (PB2-KO/Rluc virus), which is replication-incompetent because its polymerase PB2-coding region has been replaced with that of the *Renilla* luciferase reporter protein, as described previously (Ozawa et al., 2011). Because of the lack of a functional PB2, *Renilla* luciferase expression provides an indication of the efficiency of the early steps of the virus life cycle, including virus binding, internalization, nuclear import of vRNP, nuclear export of RNA, and limited translation by the PB2 protein contained in infecting vRNP complexes. A549 cells transfected with siNC or siNUP93 were infected with PB2-KO/Rluc virus. We also used amantadine as a positive control because it inhibits viral HA-mediated membrane fusion (Daniels et al., 1985). *Renilla* luciferase activity was measured at 8 h post-infection. NUP93 knockdown and amantadine treatment caused a significant reduction in *Renilla* luciferase activity (**Figure 2**). The activity in siNUP93-transfected cells was reduced by 40–60% compared with that in siNC-transfected cells. This result suggests that NUP93 is involved in the early steps of the influenza virus life cycle.

### NUP93 Does Not Affect Virus Binding, Internalization, or Nuclear Import

**FIGURE 3:**
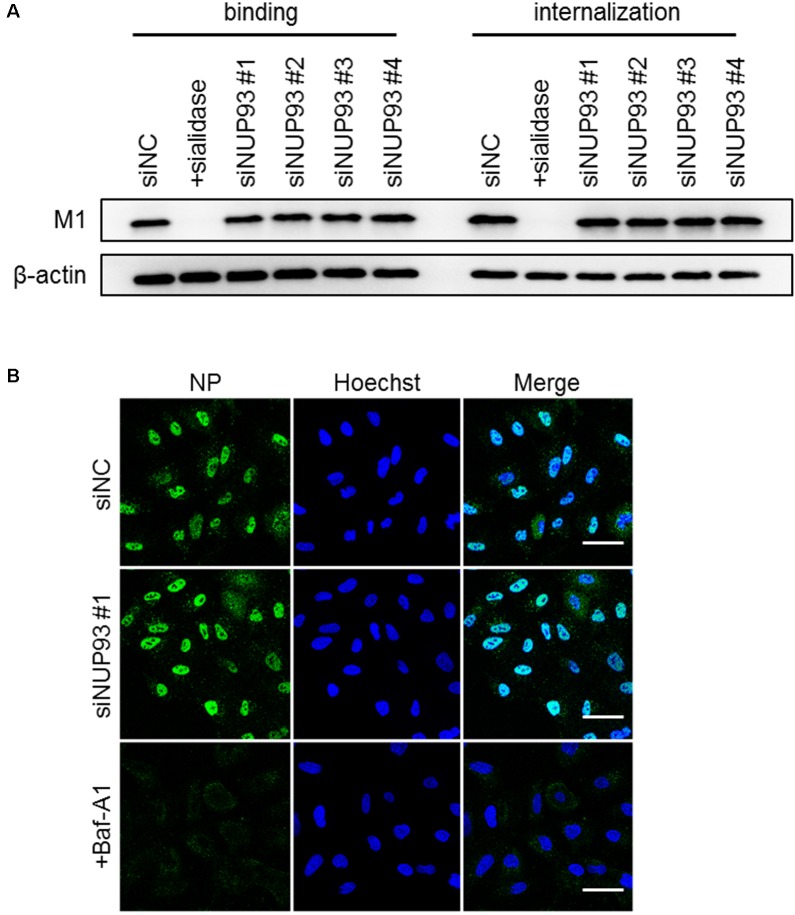
Role of NUP93 in binding, internalization, and nuclear import. **(A)** A549 cells transfected with siRNAs were infected with WSN virus at an MOI of 10. As a control, cells were pre-treated with sialidase from *Arthrobacter ureafaciens* for 8 h before the infection. M1 protein in virus particles attaching to cells, or internalized into cells, was detected by Western blotting with anti-WSN antibodies. **(B)** A549 cells transfected with siRNAs were infected with WSN virus at an MOI of 200 in the presence of CHX. Baf-A1 was used as a positive control. Cells were fixed and permeabilized at 2 h post-infection and then immunostained for NP. The images were obtained using confocal microscopy. Scale bars: 50 μm. The results are representative of at least three independent experiments.

To determine the steps in which NUP93 is involved, we assessed the effect of NUP93 knockdown on virus binding and/or internalization, the first steps of the entry process of influenza virus. To assess the efficiency of binding, A549 cells transfected with siRNAs were incubated with WSN virus at 4°C for 1 h to allow virus binding to cell surfaces while preventing the viruses from internalizing. To assess the efficiency of internalization, cells were warmed to 37°C after being incubated at 4°C, and then washed with low pH (pH 1.3) PBS to remove attached virus and detect only internalized virus. Control cells were pre-treated with sialidase to remove the sialic acid and thereby prevent virus binding and internalization. We evaluated the amount of bound or internalized virus particles by examining M1 protein levels in cell lysates. Although binding and internalization were remarkably inhibited by sialidase treatment, no such inhibition was caused by NUP93 knockdown (**Figure 3A** and Supplementary Figure 2). These findings suggest that NUP93 is not important for the virus binding and internalization steps.

We also examined the role of NUP93 in the nuclear import of vRNP. Nuclear import of vRNP occurs following membrane fusion between the internalized influenza virus and the endosome. To detect NP associated with incoming vRNP, siRNA-transfected A549 cells were infected with WSN virus at an MOI of 200 in the presence of cycloheximide (CHX) to avoid *de novo* synthesis of NP. Baffilomycin A1 (Baf-A1), which is a known inhibitor of the nuclear import of vRNP (Yeganeh et al., 2015), was used as a positive control. At 2 h post-infection, the cells were fixed, and incoming NP was detected with anti-NP antibodies. siNUP93 #1 did not cause a notable change in the localization of NP (**Figure 3B**). Similarly, the other three siNUP93s did not cause any substantial change (data not shown). Therefore, NUP93 does not appear to be involved in the nuclear import of vRNP, even though NUP93 is part of the nuclear pore complex.

### NUP93 Knockdown Downregulates the Nuclear Export of Viral RNA

**FIGURE 4:**
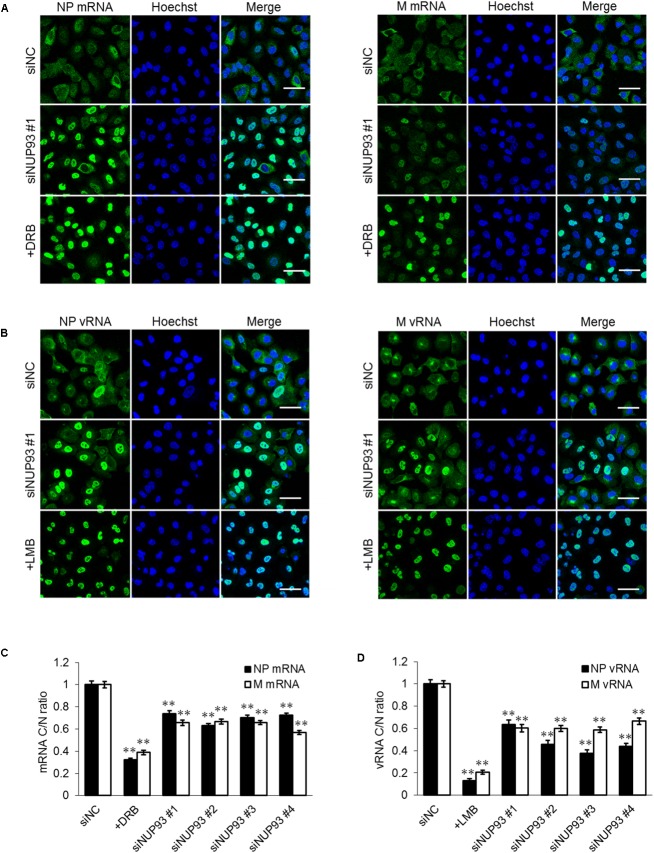
NUP93 knockdown causes accumulation of viral RNA in the nucleus. **(A,B)** A549 cells transfected with siRNAs were infected with WSN virus at an MOI of 10. Cells were fixed at 8 h post-infection and **(A)** NP/M mRNAs or **(B)** NP/M vRNAs were detected by fluorescence *in situ* hybridization. The images were obtained using confocal microscopy. Scale bars: 50 μm. **(C,D)** Cytoplasmic to nuclear ratio (C/N) of **(C)** mRNA and **(D)** vRNA at 8 h post-infection was determined in 200 cells for each condition. Values are means ± SE. ^∗∗^*p* < 0.01 (one-way ANOVA followed by Dunnett’s test). The results are representative of at least three independent experiments.

Next, we attempted to verify the importance of NUP93 in the nuclear export of influenza virus RNA. We analyzed the localization of influenza virus mRNA by using FISH. A549 cells were transfected with siRNAs, and then infected with WSN virus at an MOI of 10. As a control, cells were infected with virus in the presence of DRB, which inhibits the nuclear export of influenza virus mRNA (Killip et al., 2014). At 8 h post-infection, the cells were fixed, and the localization of the NP and M mRNAs was visualized by using specific RNA probes. DRB treatment led to an obvious accumulation of mRNA in the nucleus (**Figure 4A**). siNUP93 #1 also caused accumulation of both NP and M mRNA compared to siNC, although this effect was not as remarkable as that caused by DRB (**Figure 4A**). Similar results were obtained with the three other siNUP93s (data not shown). To confirm the change in localization, mRNA cytoplasmic/nuclear ratios (C/N ratios) were calculated based on the intensity of fluorescence. The C/N ratios of the NP and M mRNAs in siNUP93-transfected cells were reduced by 25–40% compared with those in the siNC-transfected cells (**Figure 4C**). We then examined whether the localization of the vRNA was also altered by NUP93 knockdown. LMB, which is an inhibitor of the nuclear export of vRNA (Kudo et al., 1998), caused notable accumulation of vRNA in the nucleus. vRNA accumulation in the nucleus was also observed in response to NUP93 knockdown (**Figure 4B**). Transfection with the other three siNUP93s caused similar accumulation of vRNA (data not shown). Furthermore, a reduction in the C/N ratio was confirmed quantitatively (**Figure 4D**). These results indicate that NUP93 contributes to the nuclear export of influenza virus RNA.

### Effect of NUP93 on Host mRNA Nuclear Export

**FIGURE 5:**
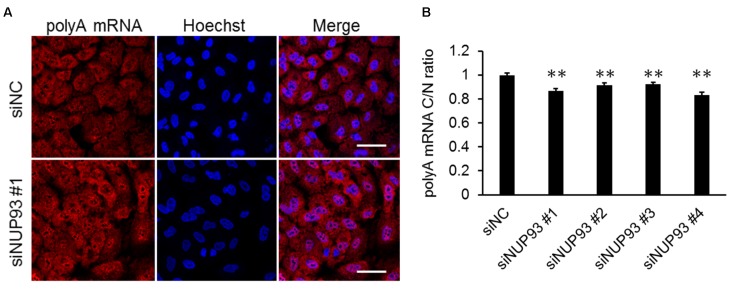
Effect of NUP93 on host mRNA nuclear export. **(A)** siRNA-transfected cells were fixed and polyA mRNA was detected by fluorescence *in situ* hybridization. The images were obtained using confocal microscopy. Scale bars: 50 μm. **(B)** Cytoplasmic to nuclear ratio (C/N) of polyA mRNA was determined in 200 cells for each condition. Values are means ± SE. ^∗∗^*p* < 0.01 (one-way ANOVA followed by Dunnett’s test). The results are representative of at least three independent experiments.

Lastly, we investigated whether NUP93 was also essential for the nuclear export of host mRNAs. The localization of host polyA RNA was analyzed by FISH. siRNA-treated A549 cells were fixed and host mRNAs was visualized by using an oligo dT probe. The C/N ratio of the host mRNAs was slightly but significantly reduced by NUP93 knockdown (**Figure 5**). This result suggests that NUP93 is involved in not only the nuclear export of viral RNA but also that of host mRNA.

## Discussion

In the present study, we showed that the host factor NUP93 was important for influenza virus replication and was required for the nuclear export of virus RNA. Although our previous interactome analysis using HEK293 cells suggested that NUP93 was involved in virus replication, it was not known whether NUP93 was also involved in virus replication in other cell types, such as respiratory epithelial cells, which influenza viruses normally target. Here, we found that NUP93 downregulation, mediated by siRNAs, reduced virus replication in human lung epithelial cells (i.e., A549 cells). Therefore, NUP93 likely plays important roles in actual virus infection of humans.

Moreover, we found that NUP93 is involved in the early steps of influenza virus replication other than virus binding and internalization. The reduction in virus replication and in the efficiency of the early steps of the virus life cycle was probably caused by the effect of siNUP93 on viral RNA transcription/replication or on its nuclear transport. Given that NUP93 is part of the nuclear pore complex, in the present study, we focused on analyzing its role in the nuclear transport of viral RNA.

NUP93 is part of one of the major subcomplexes of the nuclear pore complex and is responsible for the correct assembly of the nuclear pore complex (Grandi et al., 1997; Sachdev et al., 2012). Once influenza viruses infect host cells, vRNP is released into the cytoplasm following membrane fusion between the viral envelope and the endosomal membrane. The released vRNP must be transported into the nucleus, because influenza virus transcription and replication occur there. Therefore, we first asked whether the reduction in virus replication in NUP93-depleted cells was the result of inhibition of vRNP nuclear import caused by abnormal assembly of nuclear pore complexes. We found that the efficiency of the nuclear import of vRNP was not alter significantly in NUP93-depleted cells, suggesting that vRNPs are imported into the nucleus via an NUP93-independent pathway. Alternatively, the function of the nuclear pore complex as a barrier to limit nuclear transport may be lost by NUP93 depletion, allowing vRNPs to get into the nucleus without restriction.

After the nuclear import of vRNPs, *de novo* RNA synthesis occurs. Newly synthesized virus mRNA and vRNA go through the nuclear pore complex again and into the cytoplasm. NUP93 knockdown resulted in the accumulation of virus RNA in the nucleus. Hence, NUP93 must be required for efficient viral mRNA and vRNA export from the nucleus to the cytoplasm. This result is consistent with the fact that NUP62, which is recruited to the nuclear pore complex by NUP93 (Sachdev et al., 2012; Chug et al., 2015), is also essential for virus replication and is involved in viral RNA export from the nucleus (Morita et al., 2013; Munier et al., 2013). Although the mechanism of influenza RNA nuclear export has not been elucidated completely (Larsen et al., 2014), it has been reported that the host nuclear export factor Nxf1 is responsible for influenza virus mRNA export (Hao et al., 2008). An interaction between Nxf1 and FG Nups, nucleoporins with clustered repeats of FG (phenylalanine-glycine), is required for mRNA nuclear export through the nuclear pore complex (Santos-Rosa et al., 1998; Sträßer et al., 2000; Terry and Wente, 2007). NUP93 is not considered an FG Nup because it lacks FG domains. Therefore, NUP93 probably does not interact with Nxf1 directly. However, in the process of forming the nuclear pore complex, NUP93 interacts with several nucleoporins including FG Nups, such as NUP62, NUP58, and NUP54 (Chug et al., 2015). We suggest that NUP93 regulates mRNA nuclear export indirectly via its interaction with FG Nups. Nuclear export of vRNA is mediated by Crm1 (Neumann et al., 2000; Elton et al., 2001). Nucleoporins, which make up the nuclear pore complexes with NUP93, also play important roles in the Crm1-dependent nuclear export pathway (Oka et al., 2010; Port et al., 2015), although a direct interaction between NUP93 and Crm1 has not been reported. Correct nuclear pore complex assembly, mediated by NUP93, appears to be required for the proper interaction between Crm1 and the nucleoporins.

Here, we showed that NUP93 was also involved in the nuclear export of host mRNAs. However, the effect of NUP93 depletion on the nuclear export of host mRNA was moderate compared to that on the nuclear export of viral RNA. This observation suggests that there might be other host factors that share functional redundancy with NUP93 with respect to host mRNA nuclear export.

It was previously reported that the lipid mediator Protectin D1 inhibited the nuclear export of virus RNA specifically, without severely affecting host mRNA nuclear export (Morita et al., 2013). This finding suggests that there is a pathway, through which virus RNA is exported to the cytoplasm, that differs from the pathway for the nuclear export of host mRNA. Such a virus-specific nuclear export pathway could be a target for an antiviral drug. A deeper understanding of how virus RNA is exported from the nucleus will thus help in the development of new antiviral drugs.

## Author Contributions

YF designed the study and performed the experiments. YF, SY, and YK analyzed the data and wrote the manuscript. All the authors reviewed and approved the manuscript.

## Conflict of Interest Statement

YK has received speaker’s honoraria from Toyama Chemical and Astellas Inc.; has received grant support from Chugai Pharmaceuticals, Daiichi Sankyo Pharmaceutical, Toyama Chemical, Tauns Laboratories, Inc., Otsuka Pharmaceutical Co., Ltd., and Denka Seiken Co., Ltd.; and is a co-founder of FluGen. The remaining authors declare that the research was conducted in the absence of any commercial or financial relationships that could be construed as a potential conflict of interest.
