# Supplementary material for: Host Factor Nucleoporin 93 Is Involved in the Nuclear Export of Influenza Virus RNA
Source: Front Microbiol. 2018 Jul 24;9:1675. doi: 10.3389/fmicb.2018.01675 (PMC6066526; doi:10.3389/fmicb.2018.01675)
Supplement: Supplementary file 1 [file Data_Sheet_1.DOCX]

**Supplementary Material**

**Host Factor Nucleoporin93 Is Required for**

**The Nuclear Export of Influenza Virus RNA**

**Yuri Furusawa, Shinya Yamada, Yoshihiro Kawaoka***

***Correspondence:** Dr. Yoshihiro Kawaoka: yoshihiro.kawaoka@wisc.edu


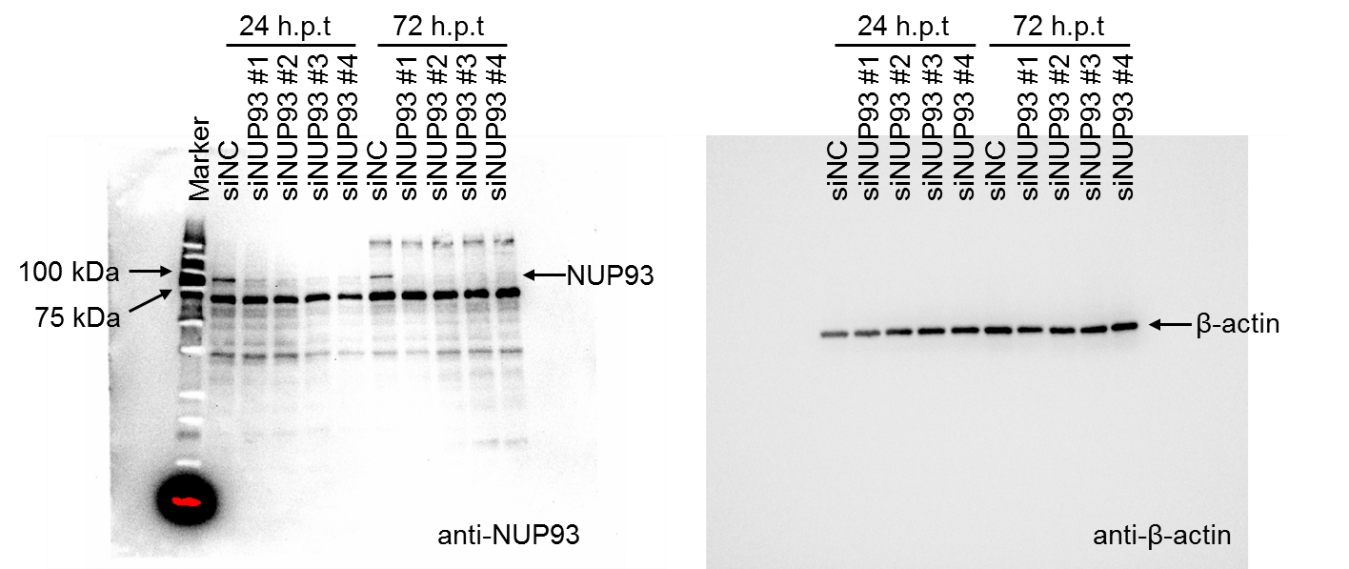


**Supplementary Figure 1.** Full blot images of Figure 1A


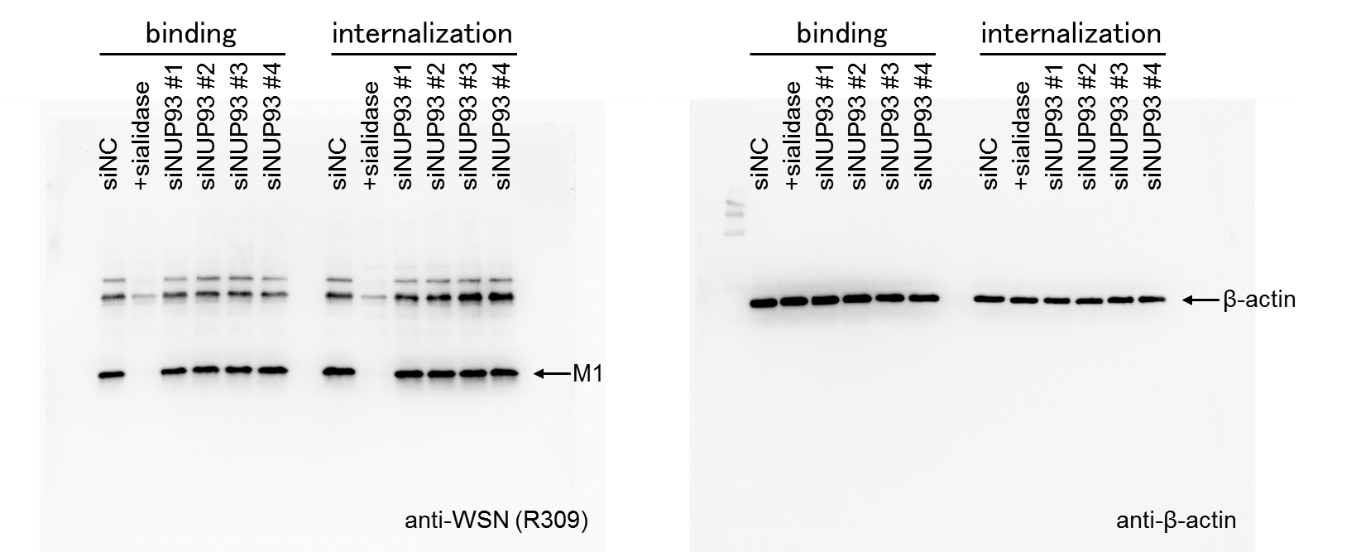


**Supplementary Figure 2.** Full blot images of Figure 3A
